# Supplementary material for: Transcriptional pathobiology and multi-omics predictors for Parkinson’s disease
Source: bioRxiv. 2024 Jun 21:2024.06.18.599639. Preprint. [Version 1] doi: 10.1101/2024.06.18.599639 (PMC11212969; doi:10.1101/2024.06.18.599639)
Supplement: Supplement 8 [file NIHPP2024.06.18.599639v1-supplement-8.pdf]

bioRxiv preprint doi: https://doi.org/10.1101/2024.06.18.599639; this version posted June 21, 2024. The copyright holder for this preprint (which was not certified by peer review) is the author/funder, who has granted bioRxiv a license to display the preprint in perpetuity. It is made available under aCC-BY 4.0 International license.

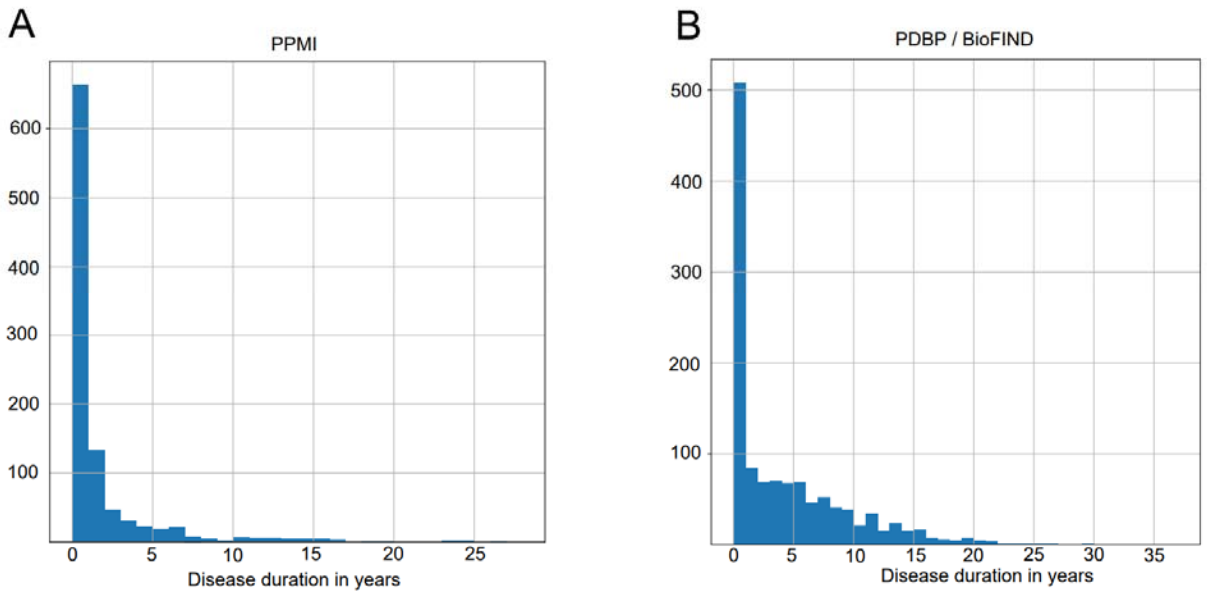

Fig S1. The distributions of disease duration at enrollment in discovery (A) and replication (B) cohort.

bioRxiv preprint doi: https://doi.org/10.1101/2024.06.18.599639; this version posted June 21, 2024. The copyright holder for this preprint (which was not certified by peer review) is the author/funder, who has granted bioRxiv a license to display the preprint in perpetuity. It is made available under aCC-BY 4.0 International license.

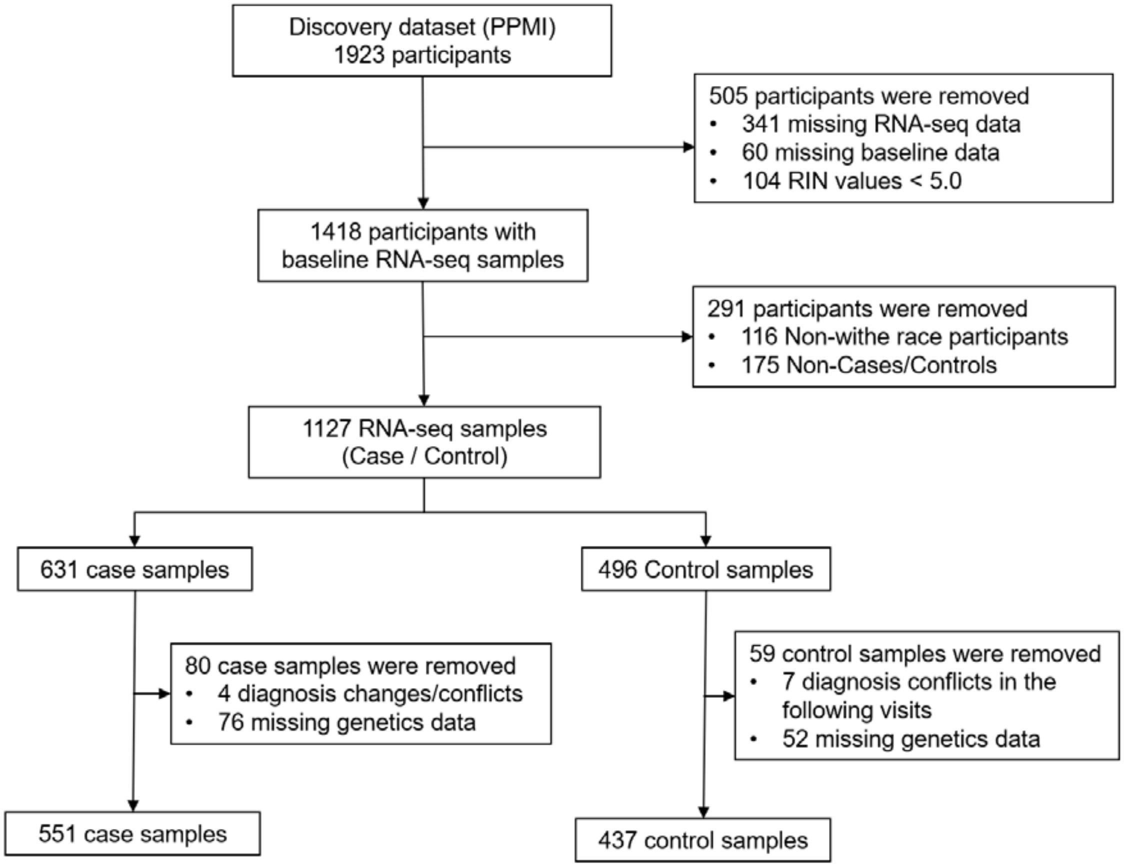

Fig S2. The steps on the discovery dataset.

bioRxiv preprint doi: https://doi.org/10.1101/2024.06.18.599639; this version posted June 21, 2024. The copyright holder for this preprint (which was not certified by peer review) is the author/funder, who has granted bioRxiv a license to display the preprint in perpetuity. It is made available under aCC-BY 4.0 International license.

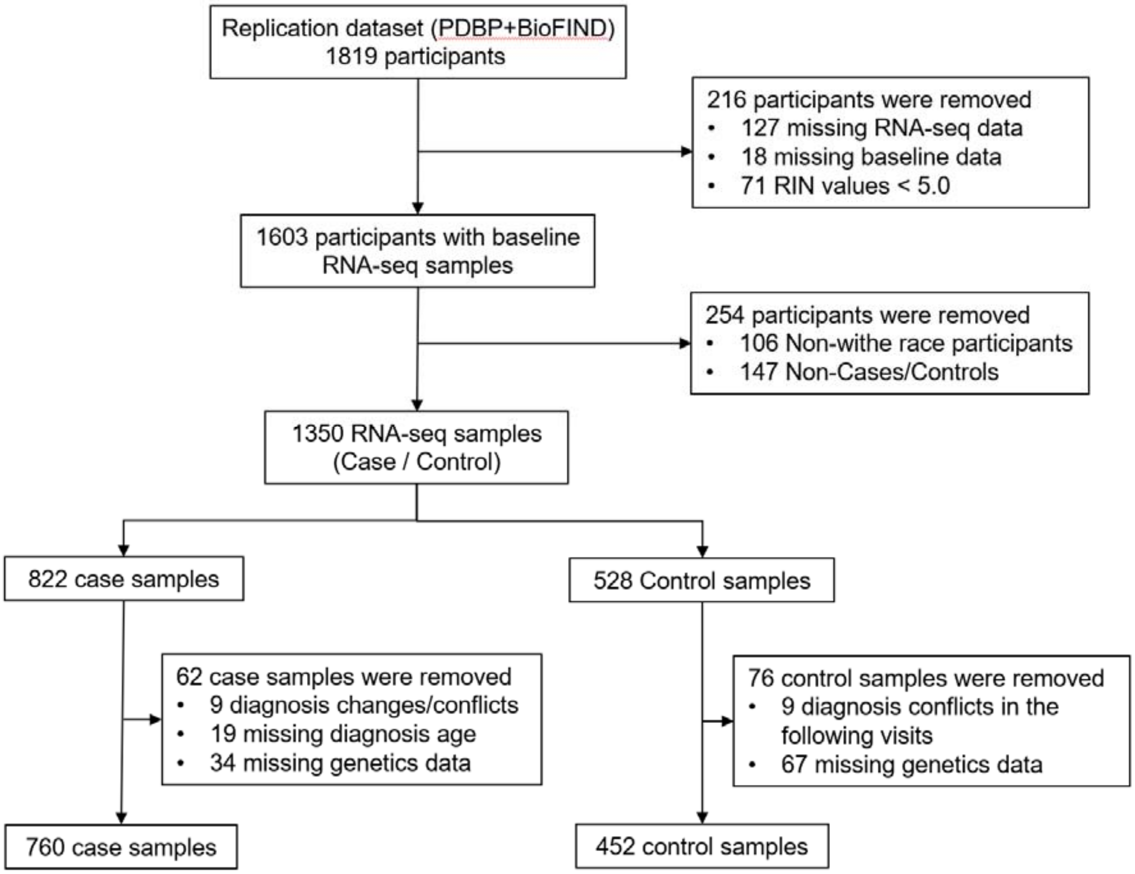

Fig S3. The steps on the replication dataset.

bioRxiv preprint doi: https://doi.org/10.1101/2024.06.18.599639; this version posted June 21, 2024. The copyright holder for this preprint (which was not certified by peer review) is the author/funder, who has granted bioRxiv a license to display the preprint in perpetuity. It is made available under aCC-BY 4.0 International license.

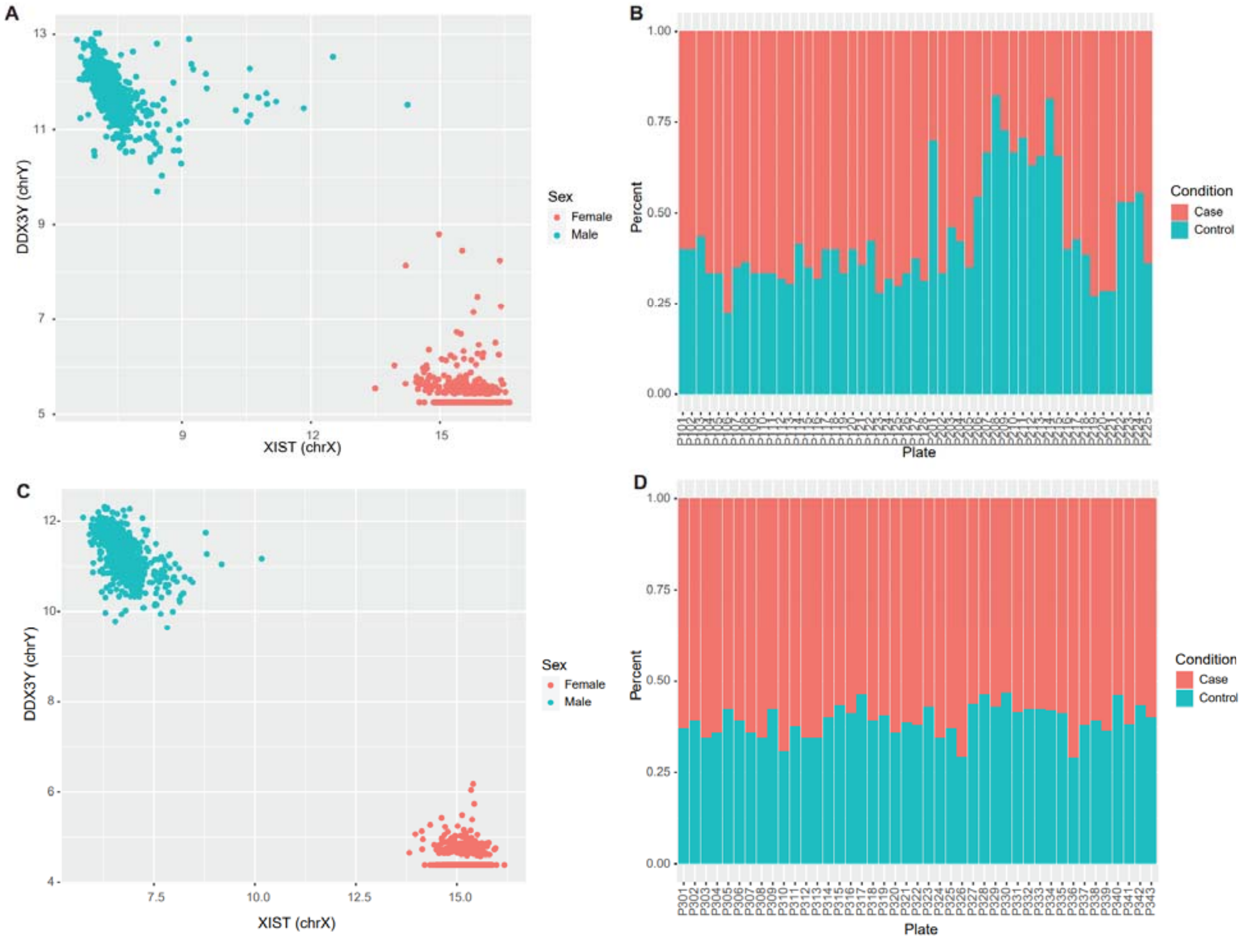

Fig S4. Data quality control assessments in the discovery and replication datasets. (A, B) Sex check and the samples distributions on the plate of discovery dataset. (C, D) Sex check and the samples distributions on the plate of replication dataset.

bioRxiv preprint doi: https://doi.org/10.1101/2024.06.18.599639; this version posted June 21, 2024. The copyright holder for this preprint (which was not certified by peer review) is the author/funder, who has granted bioRxiv a license to display the preprint in perpetuity. It is made available under aCC-BY 4.0 International license.

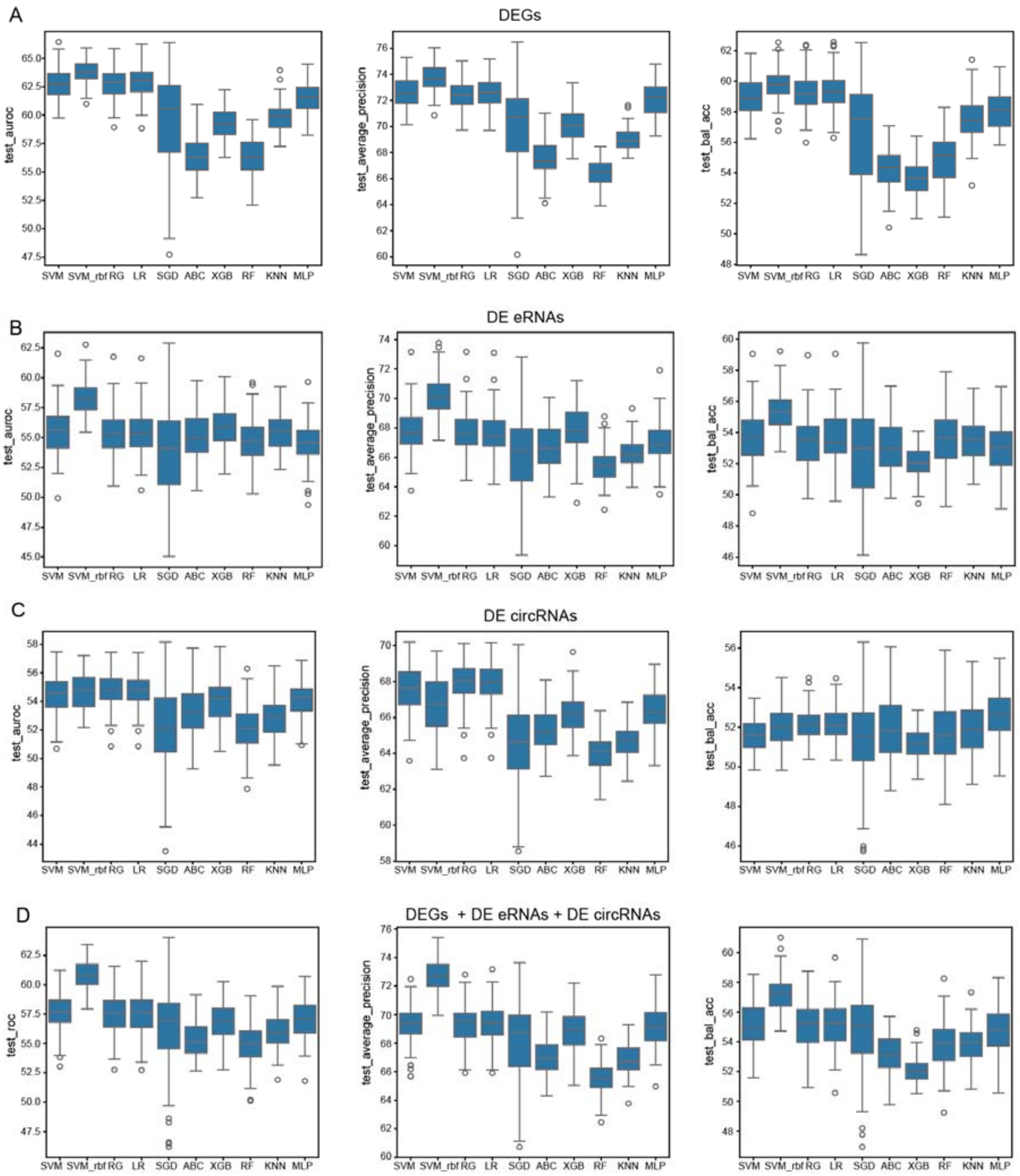

Fig S5. The AUROC, average precision and balanced accuracy values of the models using DEGs, DE eRNAs, or DE circRNAs as features.
